# Retraction: NANOG regulates epithelial mesenchymal transition and chemoresistance in ovarian cancer

**DOI:** 10.1042/BSR20160247_RET

**Published:** 2025-07-17

This article is being retracted from *Bioscience Reports* at the request of the Editor-in-Chief and the Editorial Board. This follows the receipt of a notification from a reader, alerting the Editorial Board to various duplications that appear to be present within this manuscript, and between this article and two others by different authors. Specifically, this includes:

- Overlapping sections of Figure 5C siRNA-Nanog/SKOV-3 and 5D siRNA-Nanog/SKOV-3 panels
- Overlapping sections of Figure 5C siRNA-Nanog/OV2008 and 5D siRNA-Nanog/OV2008 panels
- Overlapping sections of Figure 5D si-RNA-Renilla Luc. with sections from Figure 4B FHC miR-597–5p antagomir Migration and Invasion panels of Li et al. (2019) DOI: 10.3389/fonc.2019.00495
- Duplicate images between the Figure 5A OE-FF Luc. /T80 panel and the Figure 4B FHC Mock/Migration panel of Li et al. (2019) DOI: 10.3389/fonc.2019.00495
- Duplicate images between the Figure 5B OE-FF Luc. /T80 panel and the Figure 4B FHC Mock/Invasion panel of Li et al. (2019) DOI: 10.3389/fonc.2019.00495
- Duplicate images between the Figure 5C siRNA-Nanog/OV2008 panel and the Figure 4E LoVo miR-597–5p mimic/Migration panel of Li et al. (2019) DOI: 10.3389/fonc.2019.00495
- Duplicate images between Figure 5A OE-Nanog/T80 and the Figure 4B Mock/miR-441 mimic panel from Yang et al. (2019) DOI: 10.1590/1414-431X20197546

The Editorial Office also identified similarities between the Figure 5B OE-Nanog/T80 panel and the Figure 4B Mock/miR-376c-mimic panel from Yang et al. (2019) DOI: 10.1590/1414–431X20197546.

The authors were contacted regarding the concerns raised and cooperated with our investigation, providing various data pertaining to Figure 5. However, the data did not alleviate concerns. Given the extent of the issues raised, the Journal has lost confidence in the validity of the data, and the Editorial Board stands by the decision to retract. The authors disagree to the retraction.

